# Antiatherogenic Properties of Acetone Extract of *Alpinia zerumbet *Seeds

**DOI:** 10.3390/molecules17066237

**Published:** 2012-05-25

**Authors:** Jamnian Chompoo, Atul Upadhyay, Shinichi Gima, Masakazu Fukuta, Shinkichi Tawata

**Affiliations:** 1 Department of Bioscience and Biotechnology, The United Graduate School of Agricultural Science, Kagoshima University, Kagoshima 890-0065, Japan; Email: mcmine56@yahoo.com (J.C.); atul616@yahoo.com (A.U.); 2 Instrumental Research Center, University of the Ryukyus, Senbaru 1, Nishihara-cho, Okinawa 903-0213, Japan; Email: shingima@lab.u-ryukyu.ac.jp; 3 Department of Bioscience and Biotechnology, Faculty of Agriculture, University of the Ryukyus, Senbaru 1, Nishihara-cho, Okinawa 903-0219, Japan; Email: msfukuta@agr.u-ryukyu.ac.jp

**Keywords:** *Alpinia zerumbet*, acetone extracts, atherosclerosis, LDL oxidation, cholest-4-ene-3,6-dione

## Abstract

Oxidation of low-density lipoprotein (LDL) is the principal risk factor for the development of atherosclerosis. In this study, we used several methods to investigate the ability of the acetone extract from rhizomes, stems, leaves, flowers, pericarps and seeds of *Alpinia zerumbet* to inhibit atherosclerosis *in vitro*. The seed extract had the strongest activity against tyrosinase, pancreatic lipase (PL), 15-lipoxygenase (15-LO) and LDL oxidation activities (IC_50_ = 2.30 ± 0.02, 5.00 ± 0.07, 1.29 ± 0.07 and 15.40 ± 0.86 µg/mL, respectively), amongst all different parts. It also had similar effects to the positive controls. Most of the extracts showed partial agonistic properties towards estrogenic activity. Cholest-4-ene-3,6-dione, a steroid present only in the seed extract seems to be the compound responsible for these activities. The results showed that cholest-4-ene-3,6-dione had similar ability to curcumin and quercetin against PL and LDL oxidation (IC_50_ = 19.50 ± 1.17 and 16.12 ± 1.43 µg/mL, respectively). Furthermore, cholest-4-ene-3,6-dione (IC_50_ = 34.21 ± 1.31 µg/mL) had higher inhibition against 15-LO than quercetin (IC_50_ = 54.79 ± 1.12 µg/mL).

## 1. Introduction

Atherosclerosis is an inflammatory disease due to accumulation of cholesterol and triglycerides in blood plasma. The increase in low-density lipoprotein (LDL) content is one of the principal risk factors for atherosclerosis [1]. The process begins by accumulation of lipids within the artery wall [2]. Besides LDL, several other enzymes have been proposed to have direct or indirect effect on atherosclerosis. These include tyrosinase, pancreatic lipase (PL), and15-lipoxygenase (15-LO).

The copper containing protein ceruloplasmin has been found to stimulate LDL oxidation [3]. Tyrosinase is a polyphenolase enzyme containing copper; therefore, it is believed that inhibition of tyrosinase may prevent oxidation of LDL, thereby inhibiting atherosclerosis. PL plays a key role in the efficient intestinal digestion of triglycerides, which in turn is absorbed from the small intestine to the erythrocytes resulting in hyperlipidemia [4]. Therefore, if the digestion of triglycerides is blocked, atherosclerosis could be prevented. Furthermore, when plasma triglycerides are low, HDL cholesterol levels tend to be high. HDL opposes atherosclerosis directly, by removing cholesterol from foam cells by inhibiting the oxidation of LDL [5]. 15-LO has been suggested to be a mediator of oxidation of LDL. The 15-LO form hydroperoxy derivatives of linoleic acid and arachidonic acid and is induced in atherosclerotic plaques [6]. On the other hand, estrogens have potentially beneficial effects such as reducing LDL, increasing HDL, facilitating nitric oxide-mediated vasodilatation, and inhibiting the response of blood vessels to injury and the development of atherosclerosis. Therefore, increase in estrogenic activity prevents from atherosclerosis.

*Alpinia zerumbet* (Family Zingiberaceae) is a perennial ginger growing widely in the subtropics and tropics. It is used in folk medicine for its anti-inflammatory, bacteriostatic, and fungistatic properties [7]. The essential oil extracted from its leaves obsessed myorelaxant and antispasmodic actions in rat ileum [8]. We have reported the antioxidant activity and phenolic contents in flowers and seeds of the plant [9]. We have isolated active compounds from the rhizomes of *A. zerumbet* against HIV-1 integrase and neuraminidase enzymes [10]. Recently, we have reported the inhibitory effects of this plant on advanced glycation end products formation [11].

In this study, we chose acetone as the solvent for extracting the steroidal compounds from six different parts of *A. zerumbet *to test the atherosclerotic properties of the different extracts. We observed the inhibition of LDL oxidation and determined the estrogenic activity of different extracts and steroidal compounds. Furthermore, we probed the enzymatic activities of tyrosinase, PL and 15-LO. Finally, we identified the steroids present in different parts of the extracts and investigated their inhibition against these enzymes.

## 2. Results and Discussion

### 2.1. GC-MS Analysis

The steroidal compounds in different parts of *A. zerumbet* were identified using GC-MS by comparing their retention times and mass fragmentation pattern with a MS library. The amount of steroidal compounds analysed in crude extracts from the different parts of *A. zerumbet* are shown in Table 1. The samples contained fourteen steroids, two in the rhizome and seed, three in the flower, four in the leaf, five in the stem and eight in the pericarp. The results showed that the pericarp extract had a higher number of steroids. Sitosterol was present in higher amounts in rhizome, stem and leaf, while flower and pericarp were rich in stigmasterol. Campesterol was found in high amounts in stem and cholest-4-ene-3,6-dione was present only in the seed extract.

**Table 1:** GC-MS analysis of steroidal compounds in crude extracts from six parts of *A. zerumbet*.

Compounds	RT	Peak area (%)					
		Rhizome	Stem	Leaf	Flower	Pericarp	Seed
Cholestane	13.342	-	0.33	-	-	0.20	-
3α,7β-Dihydroxy-5β,6β-epoxycholestane	17.442	-	-	-	-	0.17	0.65
Cholest-4-ene-3,6-dione	21.067	-	-	-	-	-	1.84
Cholest-5-en-3-ol	21.125	-	-	-	0.12	-	-
Ergost-5-en-3-ol	21.133	-	-	-	-	0.29	-
Sitosterol	21.333	8.25	14.49	2.86	-	0.11	-
Cholestenone	21.567	-	-	-	-	0.96	-
5α-Ergost-8(14)-ene	21.758	-	-	0.49	-	-	-
9,19-Cyclolanostan-3-ol	21.983	-	-	0.38	-	-	-
Cholest-4-en-3-ol	22.075	-	-	-	-	0.27	-
Stigmasterol	22.225	-	2.70	-	1.46	1.72	-
Cholest-8-ene-3,6-diol	22.725	0.28	0.16	0.16	-	-	-
4,22-Stigmastadiene-3-one	22.750	-	-	-	-	0.25	-
Campesterol	24.067	-	1.55	-	0.35	-	-

RT is retention time, in min.

### 2.2. Anti-Tyrosinase Activity

**Figure 1:**
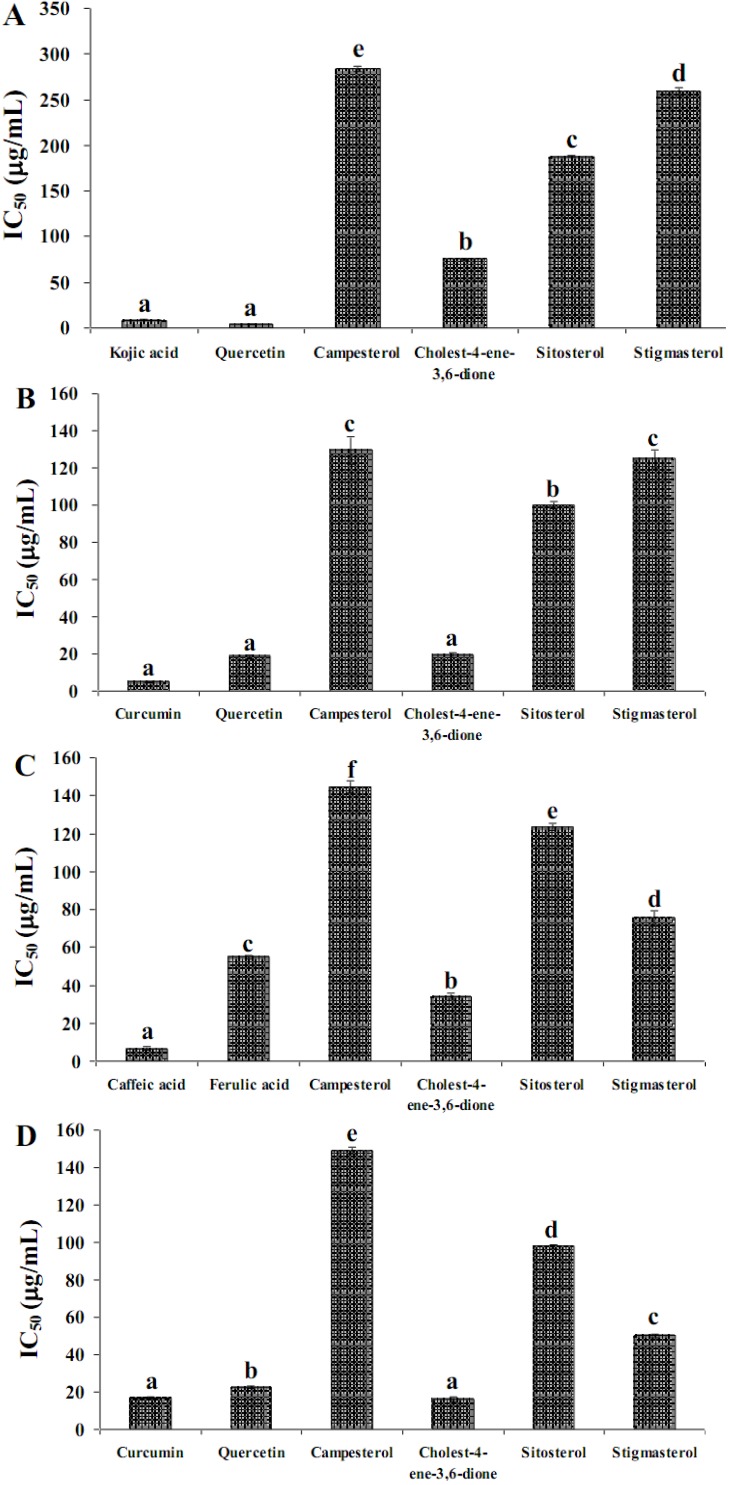
Inhibitory effect of steroidal compounds on (**A**) tryrosinase; (**B**) pancreatic lipase; (**C**) 15-lipoxygenase; and (**D**) LDL oxidation activities. Mean with the same letter on the bars are not significantly different, *p* = 0.01 (Tukey HSD). The bars represents mean ± SD (*n* = 3).

Anti-tyrosinase activity of the different parts of *A. zerumbet* is shown in Table 2. The seed extract inhibited the tyrosinase enzyme at IC_50_ = 2.30 ± 0.02 μg/mL stronger than the other parts. On carrying out inhibition with steroidal compounds, cholest-4-ene-3,6-dione (IC_50_ = 75.11 ± 0.11 μg/mL) showed higher inhibitory effect than sitosterol, stigmasterol and campesterol (IC_50_ = 187.77 ± 0.04, 259.25 ± 4.49 and 283.78 ± 3.01 μg/mL, respectively) (Figure 1A). However, cholest-4-ene-3,6-dione showed lower inhibitory effect than quercetin and kojic acid (IC_50_ = 4.92 ± 0.21 and 4.20 ± 0.24 μg/mL, respectively).

**Table 2:** Inhibitory effect of acetone extracts from various parts of *A. zerumbet *on tyrosinase, pancreatic lipase, 15-lipoxygenase and LDL oxidation activities.

Sample	50% of Inhibition (μg/mL)			
	Tyrosinase	Pancreatic lipase	15-Lipoxygenase	LDL oxidation
Rhizomes	224.53 ± 4.34 d *^1^*	277.75 ± 1.60 e	142.15 ± 1.44 c	164.90 ± 0.71 b
Stems	312.53 ± 1.43 e	747.09 ± 2.34 f	1866.75 ± 9.12 f	443.97 ± 3.64 d
Leaves	152.23 ± 1.66 b	58.88 ± 1.55 b	759.59 ± 3.13 d	507.59 ± 3.73 e
Flowers	210.33 ± 1.76 c	76.73 ± 0.82 c	1356.57 ± 0.52 e	423.43 ± 3.47 c
Pericarps	151.31 ± 0.26 b	256.30 ± 3.12 d	87.90 ± 1.60 b	515.48 ± 2.14 e
Seeds	2.30 ± 0.02 a	5.00 ± 0.70 a	1.26 ± 0.07 a	15.40 ± 0.86 a

*^1^* The data represent the means ± SD of three determinations. Values with the same letter in one column are not significantly different (*p *= 0.01) from each other.

### 2.2. Pancreatic Lipase Inhibition

The seed extract (IC_50_ = 5.00 ± 0.07 μg/mL) showed the highest inhibitory effect on PL activity as shown in Table 2. Cholest-4-ene-3,6-dione (IC_50_ = 19.50 ± 1.17 μg/mL) showed similar results to the positive controls, curcumin and quercetin (IC_50_ = 4.92 ± 0.21 and 18.60 ± 0.86 μg/mL, respectively) and it had stronger inhibitory effect than campesterol, stigmasterol and sitosterol (IC_50_ = 129.50 ± 7.36, 125.05 ± 4.76 and 99.99 ± 1.86 μg/mL, respectively) (Figure 1B).

### 2.3. 15-Lipoxygenase Inhibition

The IC_50_ result for 15-LO inhibition of the six parts of *A. zerumbet* is presented in Table 2. The highest activity towards 15-LO inhibition was observed in the seed extract (IC_50_ = 1.29 ± 0.07 μg/mL). The inhibition ability of steroidal compounds on 15-LO is shown in Figure 1C.

Cholest-4-ene-3,6-dione (IC_50_ = 34.21 ± 1.31 μg/mL) had higher inhibitory effect than campesterol, sitosterol and stigmasterol (IC_50_ = 144.72 ± 3.19, 123.34 ± 1.94 and 75.26 ± 3.94 μg/mL, respectively), while it had weaker inhibition than the positive control caffeic acid (IC_50_ = 6.43 ± 0.97 μg/mL) but stronger inhibition than ferulic acid (IC_50_ = 54.79 ± 1.12 μg/mL).

### 2.4. LDL Oxidation Inhibition

Table 2 shows 50% inhibition of various parts of *A. zerumbet* on LDL oxidation, assessed with the TBARS assay. The seed extract (IC_50_ = 15.40 ± 0.86 μg/mL) showed the highest reduction in the amount of MDA equivalents produced by copper-induced LDL oxidation compared with other parts. The inhibition ability of steroidal compounds is shown in Figure 1D. Cholest-4-ene-3,6-dione (IC_50_ = 16.12 ± 1.43 μg/mL) had higher inhibitory effect than campesterol, sitosterol and stigmasterol (IC_50_ = 149.02 ± 1.93, 97.76 ± 0.77 and 50.01 ± 0.77 μg/mL, respectively). Moreover, cholest-4-ene-3,6-dione showed significantly better activity than quercetin (IC_50_ = 22.56 ± 0.70 μg/mL), while it exhibited a similar result to curcumin (IC_50_ = 16.12 ± 1.43 μg/mL).

### 2.5. Estrogenic Activity

**Figure 2:**
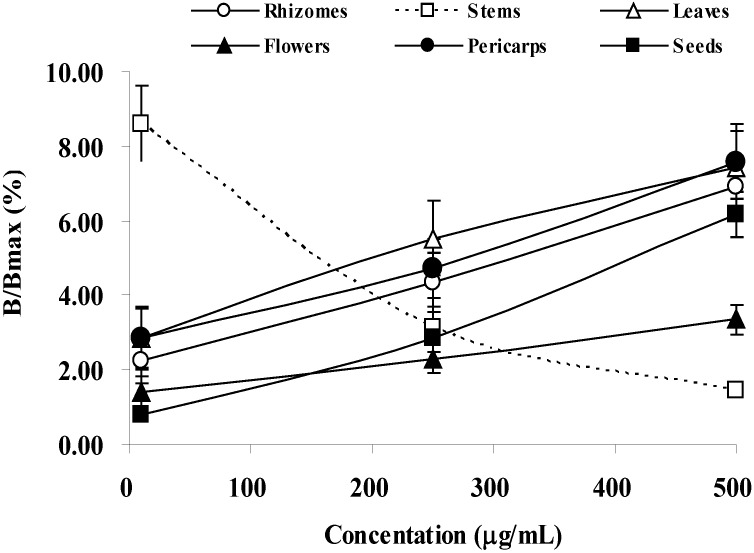
Dose response curve of different part extracts of *A. zerumbet* for estrogenic activity. Values are expressed as the mean ± SD of three replicate experiments.

The relative activity of estrogen by various concentrations of the crude extracts from different parts of *A. zerumbet* is shown in Figure 2. The results showed that the activity increases with higher concentration. Interestingly, the stem extract showed different results. At 500 μg/mL, all samples except stem showed partial agonistic properties.

### 2.6. Discussion

It is widely accepted that oxidative modification of plasma lipoproteins, particularly LDL, plays an important role in the initiation of atherosclerosis. It has been reported that terpenoids inhibit the oxidative modification of LDL *in vitro *[12]. In addition, plant sterols and stanols (phytosterols/phytostanol) are known to reduce serum LDL-cholesterol level, and food products containing these plant compounds are widely used as a therapeutic dietary option to reduce plasma cholesterol and atherosclerotic risk [13]. In this study, we investigated the inhibitory effects of six different parts and steroidal compounds containing extracts of *A. zerumbet* against atherosclerosis. For this, we explored inhibition of tyrosinase enzyme, which is a multifunctional, glycosylated and copper-containing oxidase [14] that may be related with oxidation of LDL. Furthermore, as the link between PL and atherosclerosis is well established, we checked the inhibition of PL by different extracts and steroidal compounds. We also investigated the inhibition of 15-LO since this is a lipid-oxidizing enzyme that is considered to contribute the formation of oxidized lipids in atherosclerotic lesions [15]. In addition, we also studied LDL oxidation inhibition because it leads to development of atherosclerosis. Finally, we probed the effects of *A. zerumbet* parts on estrogenic activity since there is an inverse relationship between estrogenic activity and LDL oxidation [16].

Our results indicated that seed extract had better activities amongst all of the parts. Numerous previous studies have reported similar results. For instance, Lin *et al*. [17] had reported that *A. zerumbet* seed was an effective hypolipidaemic, with amazingly potent high-density lipoprotein cholesterol (HDL-C) enhancing ability as well as low-density lipoprotein cholesterol (LDL-C) suppressant activity. It could be used as an effective hepatoprotector and anti-atherosclerosis agent. Furthermore, several reports have been published on the other potential usages of Alpinia seed. Lee *et al*. [18] revealed that seed extract of *A. katsumadai* inhibited H_2_O_2_-induced apoptosis and dose-dependently enhanced the activities of superoxide dismutase, catalase and glutathione peroxidase in Chinese hamster lung fibroblast (V79-4) cells. It had also been reported to protect neurons from ischemic damage [19]. Besides, it was found to significantly inhibit increases in Th2-type cytokine level, eosinophilia and mucus hypersecretion in the asthmatic mouse model [20]. Moreover, Mitsui *et al*. [21] reported that *A. galangal* seeds showed antiulcer activity in Shay rats.

In order to identify the active phytochemicals, we carried out GC-MS analysis of all different parts. We identified a number of steroidal compounds present in the six parts of *A. zerumbet*. Interestingly, we found cholest-4-ene-3,6-dione was present only in the seed extract. It seems that this compound may have roles in inhibiting atherosclerosis. Furthermore, sitosterol was found in higher amounts in stem, rhizome and leaf. However, it has been reported that sitosterol and campesterol could not inhibit enzymes in thecholesterol biosynthetic pathway [22] and therefore seems to have lesser effects in reducing atherosclerosis. On the other hand, stem, flower and pericarp also contained stigmasterol that has been reported to have inhibitory effects in cholesterol biosynthesis [22]. In order to investigate the efficacy of different steroids, which were present in relatively high amounts (>1% peak area) high amounts in different parts of *A. zerumbet*, we carried out tyrosinase, PL, 15-LO and LDL oxidation by pure steroidal compounds. The results showed that cholest-4-ene-3,6-dione had the highest activity in all assays, and therefore, it seems that the activity of seed extract may be due to the presence of this steroid. The potency of numerous other steroidal compounds has been reported to possess atherosclerosis inhibition properties. The adrenal steroid dehydroepiandrosterone (DHEA, 3*β*-hydroxy-5-androsten-17-one) has been shown to reduce serum LDL level in normal human [23] and inhibited the development of accelerated atherosclerosis in native hearts of a rabbit heart transplant model [24]. However, cholest-4-ene-3,6-dione has been reported to show inhibitory activity on proliferation of rat hepatic stellate cells [25] and cytotoxic activity against human cancer cell line [26].

Estrogens are widely regarded as beneficial to arterial wall health. Among the mechanisms of this benefit are antioxidant effects on LDL and the arterial wall [27]. The results on estrogenic activity reveal that all parts, except stem, had higher the activity with increasing concentration. At the concentrations used, the relative activity of five different parts to the positive control, 17-*β*-estradiol, seems to be of partial agonistic. The plausible explanation for the antagonistic effect of stem extract toward estrogenic activity is still under investigation.

Therefore, our results indicate that seed extracts can inhibit the formation of atherosclerosis by inhibiting tyrosinase, PL, 15-LO, and LDL oxidation and by partially inducing estrogenic activity. Furthermore, cholest-4-ene-3,6-dione, the steroidal compound, is found only in the seed of *A. zerumbet* that had high potential inhibition activities in all of performed experiment.

## 3. Experimental

### 3.1. General

Quercetin, L-tyrosine, caffeic andferulic acids were obtained from Wako Pure Chemical Industries, Ltd. (Osaka, Japan). Trichloroacetic acid (TCA), thiobarbituric acid (TBA), *p*-nitrophenylbutyrate (*p*-NPB), low-density lipoprotein cholesterol from human plasma (LDL), porcine pancreatic lipase (PL) and tyrosinase mushroom and cholest-4-ene-3,6-dionewere purchased from Sigma-Aldrich, Inc. (Missouri, USA). Curcumin and ethylenediaminetetraacetic acid (EDTA) were bought from Kanto Chemical Co, Inc. (Tokyo, Japan). Campesterol, sitosterol and stigmasterol were obtained from Tama Biochemical Co., Ltd. (Tokyo, Japan). 3-Morpholinopropanetetraacetic acid (MOP) was secured from Dojindo Molecular Technologies, Inc (Tokyo, Japan). EnBio RCAS for ERα was bought from Fujikura Kasei Co., Ltd. (Tokyo, Japan). 15-Lipoxygenase from soybean (15-LO) was procured from Cayman Chemical Company (Michigan, USA).

### 3.2. Plant Material and Preparation of Extracts

Rhizomes, stems, leaves, flowers, pericarps and seeds of *A. zerumbet* were collected at University of the Ryukyus, Okinawa, Japan. Twenty grams of dry samples were cut into pieces and extracted in acetone (70%) 400 mL for 24 h at room temperature. After filtration, acetone was evaporated under vacuum in a rotary evaporator at 40 °C. All the crude extracts were kept in refrigerator for further analysis.

### 3.3. GS-MS Analysis

The steroids analytical column was HP-1MS capillary column (Agilent Technologies, J&W Scientific Products, Folsom, CA, USA). Helium was used as the carrier gas at a flow rate of 1 mL/min. The temperature was programmed at 80 °C for 5 min then increased to 300 °C at the rate of 15 °C per min. The temperatures of injector and EI detector (70 eV) were 280 °C and 300 °C, respectively. Each plant extract of 2.0 μL was injected to the GC-MS [28].

### 3.4. Anti-Tyrosinase Activity Assay

Tyrosinase activity inhibition was determined by the method as described previously [29]. In brief, sample extracts were dissolved in methanol to make the different concentrations (μg/mL). The 96-well plate was set up in the following order; 120 μL of phosphate buffer (20 mM, pH 6.8), 20 μL of sample and 20 μL of tyrosinase mushroom (500 units/mL in 20 mM phosphate buffer). After incubation at 25 °C for 15 min, reaction was initiated by adding 20 μL of 0.85 mM L-tyrosine solution to each well. The enzyme activity was determined by measuring the absorbance at 470 nm using microplate reader (BioRad, Benchmark microplate reader, Hertfordshire, UK). Kojic acid and quercetin were used as positive controls. The percentage of tyrosinase inhibition was calculated as follows: 


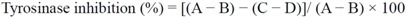


where, A = absorbance of the control with enzyme; B = absorbance of the control without enzyme; C = absorbance of the test sample with enzyme; D = absorbance of the test sample without enzyme.

### 3.5. Pancreatic Lipase Inhibition Assay

The ability of the different parts of crude extracts to inhibit PL was measured as described previously [30], with slight modifications. Briefly, an enzyme buffer was prepared by the addition of 30 μL (10 units) of PL solution (in 10 mM MOPS and 1 mM EDTA, pH 6.8) to 850 μL of Tris buffer (100 mM Tris-HCl and 5 mM CaCl_2_, pH 7.0). Then, 100 μL of the sample extracts or curcumin or quercetin was mixed with 880 μL of the enzyme buffer, and was incubated for 15 min at 37 °C. The reaction was allowed to proceed for 15 min at 37 °C after addition of 20 μL of the substrate solution (10 mM *p*-NPB in dimethyl formamide). The lipase activity was determined by measuring the hydrolysis of *p*-NPB to *p*-nitrophenol at 400 nm using microplate reader. Inhibition of the lipase activity was expressed as the percentage decrease in the OD when PL was incubated with the test compounds.

### 3.6. 15-Lipoxygenase Inhibition Assay

Enzyme inhibition was determined as described previously in borate buffer (0.2 M, pH 9.0) by measuring the increase in absorbance at 234 nm in 5 min after addition of 15-LO, using linoleic acid (134 μM) as substrate [31]. The final enzyme concentration was 500 units/mL and the test samples were dissolved in DMSO solutions. All measurements were carried out at least twice, in each instance using caffeic and ferulic acids, a well-known inhibitor of 15-LO, as positive controls. Calculations of enzyme activity were carried out as previously described, and IC_50_ values were determined by linear interpolation between the measuring points closest to 50% activity.

### 3.7. LDL Oxidation Inhibition Assay

The oxidation of LDL was investigated as described by Rattan and Arad [32]. CuSO_4_-induced oxidized-LDL generation were performed using 100 μL of LDL (220 μg/mL) incubated at 37 °C in dark with 10 μL of 55 μM CuSO_4_ and 10 μL of test extracts or curcumin and quercetin for 24 h. The reaction was stopped by adding 50 μL of 1 M EDTA and placing the sample at −20 °C for TBA reactive substance (TBARS) assay. The generation of malonyldialdehyde (MDA) equivalents during LDL oxidation was estimated by the TBARS assay using the method of Steinbrecher *et al*. [33]. LDL oxidation was carried out as described above. After oxidation, LDL was mixed with 1.5 mL of 0.67% TBA and 1.5 mL of 20% TCA. After placing samples in boiling water (100 °C) for 30 min, the reaction product was kept for 30 min at 25 °C and centrifuged for 15 min at 4 °C. The supernatants were read on a spectrophotometer at 532 nm, blank containing 220 μg/mL LDL only. The yields of MDA were used as a standard and the results were expressed as nanomoles of MDA equivalents.

### 3.8. Estrogenic Activity Assay

The estrogenic activity of six parts of crude extracts was determined by EnBio RCAS for estrogen receptorα (ERα) kit (Fujikura Kasei Co., Ltd, Tokyo, Japan, Catalog No. 7664-93-9, UN No. 2796). Briefly, a peptide containing LxxLL motif of coactivator (SRC1) was immobilized on the microplate by incubating SRC1 for 1 h at room temperature. After washing by wash buffer, 5 μL of test sample and 95 μL of ERα were added to the wells. The mixtures were incubated with shaking for 1 h at room temperature. After washing, 100 μL of detection antibody solution was added and further incubated for 30 min at room temperature. 100 μL of TMB substrate solution was added after washing and further incubated without shaking for 20 min. Absorbance was measured at 450 nm after addition of 100 μL of stop solution. 17*β*-Estradiol was used as a positive control, while DMSO was used a negative control. The estrogenic activity relative to 17*β*-estradiol, B/Bmax (%), was calculated by:


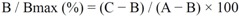


where, A = OD_450_ value of the positive control in (SRC (+) well − SRC (−) well); B = OD_450_ value of the negative control in (SRC (+) well − SRC (−) well); C = OD_450_ value of the test sample in (SRC (+) well − SRC (−) well).

### 3.9. Statistical Analysis

IC_50_ values were expressed as mean ± standard error mean by plotting the curve with percentage of inhibition versus concentrations of the individual experiments measured (*n *= 3). Statistical analysis was performed by one-way ANOVA. Upon significant difference, mean were separated using Tukey’s HSD range test at *p *= 0.01. All statistical analyses were performed using SPSS version 16.0 for Windows.

## 4. Conclusion

The acetone extract of *A. zerumbet *seeds contains several metabolites that have inhibitory effects against atherosclerosis. The present results indicate that seed extract is an efficient scavenger of free radicals and inhibition of enzymatic activities. Our preliminary studies suggest that acetone extract from seeds of *A. zerumbet *may be used to produce a natural supplement or added into health food products to prevent atherosclerosis disease. Furthermore, cholest-4-ene-3,6-dione was present only in seed acetone extract, showed the strongest inhibition activities and had no significantly difference in activity when compared with positive controls. Thus, cholest-4-ene-3,6-dione is steroidal compound that could be used to prevent LDL oxidation. This seed extract will further be studied in order to isolate and identify phytochemical compounds, involved in the ability on inhibition atherosclerotic disease.
